# Positive Relationships with Adults and Resilience to Suicide Attempt among New Mexico Hispanic Adolescents

**DOI:** 10.3390/ijerph181910430

**Published:** 2021-10-04

**Authors:** Meryn Hall, Lynne Fullerton, Dan Green, Courtney A. FitzGerald

**Affiliations:** 1 Department of Emergency Medicine, University of California, Fresno, CA 93701, USA; Meryn.Hall@ucsf.edu; 2 Department of Emergency Medicine, University of New Mexico Health Sciences Center, Albuquerque, NM 87131, USA; 3 New Mexico Department of Health, Epidemiology and Response Division, Santa Fe, NM 87505, USA; dan.green@state.nm.us; 4 Department of Pediatrics, University of New Mexico Prevention Research Center, Albuquerque, NM 87101, USA; cafitzgerald@salud.unm.edu

**Keywords:** suicide attempt, prevention, adolescent, Hispanic, resiliency, family, community

## Abstract

Suicide is a leading cause of adolescent death and has increased in recent years. The purpose of this study was to examine the effect that relationships with adults at home and in the community had on the probability of suicide attempts of Hispanic teenagers in New Mexico. Data from the 2019 New Mexico Youth Risk and Resiliency Survey were analyzed to identify the ways in which relationships with adults influenced suicide attempts among Hispanic adolescent students. The examined factors included: relationships with adults in the home and in the community and with same-age friends, and participation in hobbies or organizations outside of school. The resiliency factors were similar for male and female Hispanic students. As positive relationships with adults at home or in the community increased, the probability of suicide attempts decreased by 37–54%. Positive relationships with same-age friends were also associated with reduced suicide attempts. Community organization involvement and hobbies affected males and females differently. Adults at home and in the community can decrease the risk of suicide for Hispanic teenagers through supportive relationships. Hybrid programs focusing on adolescent health, positive communication, and academic support, which integrate adults from home and community environments, show promise in reducing suicidal thoughts and other risk behaviors.

## 1. Introduction

Suicide is the second leading cause of death among all age groups in the US from 10 to 34 years [1], and death rates among adolescents are increasing. In the 13- to 19-year-old age group, rates rose from 6.9/100,000 in 2013 to 8.8/100,000 in 2019, a 28% increase [2]. The data from the biennially conducted Youth Risk Behavior Survey (YRBS) indicate that suicide attempt rates were stable or declining during the same period [3], which suggests that the lethality of attempts may be increasing. The reasons for adolescent suicide attempts are complex, and numerous risk factors in the home, community and school settings are identified [4,5,6]. Adolescent suicidal behavior and deaths make lasting impacts on families, who may be left confused about the lack of signs prior to suicide, and who struggle with stress, anxiety and depression in the wake of losing a valued family member [7]. Friends of adolescents who die by suicide also struggle with depression and suicidal ideation [8], and the larger community is affected as well [9,10], including through potential suicide contagion [11].

New Mexico ranks seventh in the nation for suicide death for adolescents aged 13–19 years over the past 5 years (2015–2019) with a rate of 16.8 per 100,000 [12]. The adolescent suicide attempt rate in New Mexico was 9.9% in 2017 [13]. Nationally, suicide attempt rates are higher for female Hispanic (11.9%) adolescents than non-Hispanic whites (9.4%) but similar for male Hispanic (5.5%) and non-Hispanic white (6.4%) adolescents [14]. New Mexico is the only US state where Hispanics outnumber non-Hispanic whites [15].

For Hispanic teenagers of both genders, the acculturation gap—a schism between the values of parents and children—is shown to be a risk factor for suicide and self-harm behavior [16]. Discrimination stress is a risk factor specific to males, and family drug use and immigration stress are risk factors specific to females. Friendships play a somewhat unclear and complicated role in suicide risk for Hispanics [16]. The lack of meaningful social ties can lead to feelings of isolation and self-destructive behavior, including suicide attempts, among Hispanic females [17]. For Mexican-American girls, social disconnectedness from friends at school is a risk factor for suicidal ideation, especially for academically high achieving girls [18]. Friendships do not appear to have the same impact on Mexican American boys. However, social anxiety disorder is identified as a risk factor for Latino adolescents of both genders [19].

Much of the research on suicide in Hispanic or Latinx adolescents is focused on females. The lack of positive relationships with adults are identified as risk factors for Hispanic girls, including a lower perceived parental and teacher support, ref. [20] and lower sense of mutuality with their mothers [21,22,23]. For Latina suicide attempters who expressed clear intent to die, familism, or the sense that they must sacrifice themselves and their happiness for the sake of their family, was a commonly expressed sentiment [24]. Additional Latina-specific risk factors identified from a longitudinal analysis of YRBS data include experiencing physical fighting or bullying at school, forced sexual intercourse, four or more lifetime sexual partners, and drug use [25,26]. For Hispanic teenagers in New Mexico of both sexes, food insecurity and being born outside of the US were risk factors, and for girls, speaking a language other than English at home was associated with suicide attempts [27].

Less is known about which factors may help protect against suicide for Hispanic teens. Studies not specific to Hispanic teens, i.e., across all ethnicities, indicated that the support of parents and friends was protective against suicidal ideation and suicidal behaviors [6]. For Hispanics of both sexes, a strong sense of ethnic identity was protective against suicidal ideation and suicide attempt, and a strong ethnic identity moderated the increased risk of suicidal behaviors associated with having an incarcerated family member [28]. Strong cultural connections, which seem to correlate with strong mother–daughter relationships, was associated with a reduced risk of suicide attempts for female Hispanics [29]. For New Mexican Hispanic teenagers, positive relationships with adults in the school environment are shown to be protective against past-year suicide attempts for both genders [27].

The purpose of this study was to examine past-year suicide attempt prevalence among adolescent Hispanic students in the context of relationships with adults at home and in the community that may prove protective against suicide. We hypothesized an ordinal relationship between the magnitude of positive relationships with adults outside the school environment and the probability of suicide attempt, as well as ordinal relationships between community engagement in the form of extracurricular activities and hobbies and the probability of suicide attempt. We further hypothesized, based on the literature reviewed above, that the probability ratios associated with relationships with parents and with friends would be higher (more protective) among female than male Hispanic adolescents.

## 2. Materials and Methods

### 2.1. Instrumentation

The Youth Risk Behavior Survey (YRBS) is administered by the Centers for Disease Control (CDC) at US public high schools biennially to measure behaviors associated with common causes of injury and illness among US adolescents. Participating states can modify the national YRBS survey by adding or subtracting a small number of questions. New Mexico’s version of the YRBS, the New Mexico Youth Risk and *Resiliency* Survey (NM-YRRS), adds several questions regarding resilience not included in the YRBS.

### 2.2. Procedure

The NM-YRRS is administered during high school class time, and student participation is anonymous and voluntary. Detailed methods for national YRBS administration are published elsewhere [30]. New Mexico uses a computer program to randomly select schools, and sample sizes within each school. School administrators choose the date of the survey administration and select an appropriate class or class period that gives each student an equal chance of participating. The sample size varies from year to year based on the estimated response rates and current high school enrollment numbers.

### 2.3. Participants

Study participants were students who completed the 2019 NM-YRRS, identified themselves as Hispanic, and answered the question about whether or not they had attempted suicide within the preceding year. Hispanic identity was based on responses to two questions. The first was the question, “Which of these groups best describes you?” with the options: American Indian or Alaska Native; Asian; Black or African American; Hispanic or Latino; Native Hawaiian or other Pacific Islander; White). Of the 19,227 students who submitted surveys, 18,364 (95.5%) responded to this race/ethnic identity question. Of those, 8759 (50.5%) identified as Hispanic. The second ethnicity question was “Are you Hispanic or Latino?” There were 11,840 positive responses to this question, with a response rate of 98.9%. Combining data from the two questions resulted in a sample of 11,887 participants who indicated Hispanic ethnicity. Twenty-four people did not answer the question about gender and an additional 1822 did not respond to the question about past-year suicide attempt, leaving a final sample size of 10,041.

### 2.4. Outcome and Predictor Variables

Past-year suicide attempt was assessed by the question “During the past 12 months, how many times did you actually attempt suicide?” (0 times; 1 time; 2 or 3 times; 4 or 5 times; 6 or more times). We recoded this to be binary (zero attempts; one or more attempts).

Seven predictor variables characterized students’ relationships with adults at home and in the community, and with friends their own age. Two additional variables described students’ engagement in group activities and hobbies in the community (outside of home and school). All predictor variables were measured using an ordinal assessment of agreement with statements: “How true do you feel the following statements are for you?” Responses were measured on a Likert scale that included “not true at all,” “a little true,” “pretty much true,” and “very much true.” (Variables related to relationships with school adults, and to school activities, were addressed in a separate paper [27].)

Statements related to adults at home included: “In my home there is a parent or some other adult who is interested in my schoolwork,” “In my home there is a parent or some other adult who believes I will be a success,” and “When I am not home, one of my parents or guardians knows where I am and who I’m with.” Statements regarding community adults were “Outside of my home and school there is an adult who really cares about me” and “Outside of my home and school there is an adult who tells me when I do a good job.” Statements regarding peers included “I have a friend my own age who really cares about me” and “I have a friend my own age who helps me when I am having a hard time.”

Community activities were assessed using two statements: “Outside of my home and school, I am part of clubs, sports teams, church/temple or other group activity” and “Outside of my home and school, I am involved in music, art, literature, sports or a hobby.”

Age, school grades, and parental education were included as control variables. School grades were measured by the question, “During the past 12 months, how would you describe your grades in school?” (mostly As; mostly Bs; mostly Cs; mostly Ds; and mostly Fs). The question was reverse-coded such that increasing values represented better grades. The participants’ mother’s and father’s education were recorded as the highest level of schooling completed, with options “completed grade school or less; some high school; completed high school; some college; completed college; graduate or professional school; not sure.” The responses were coded as ordinal with the last option coded as missing.

### 2.5. Data Analysis

All data analyses were conducted using survey commands available in Stata version 14 [31]. Descriptive statistics, stratified by sex, included distributions of responses for each posited predictor variable and the prevalence of past-year suicide attempt for each response level. Probability ratios and 95% confidence intervals were used to examine bivariable relationships between predictor variables and suicide attempts. Relationships were recalculated while controlling for possible confounders: student age, usual grades in school, mother’s education, and father’s education. Multivariable logistic regression was conducted to control possible confounders and model sex-specific risk factors for past-year suicide attempt. Model was manual reverse stepwise regression, with the initial full model including all predictor variables with *p* < 0.1 in binary analysis, age, school grades, mother’s education, and father’s education. A type one error rate of 0.05 was the predetermined threshold for statistical significance.

## 3. Results

Among 10,041 Hispanic students, 9.5% reported one or more suicide attempts in the preceding 12 months; the prevalence among girls (11.6%) was higher than for boys (7.4%) (*p* < 0.0001). These percentages were similar to non-Hispanic children in the same sample, of whom 10.1% reported one or more past-year suicide attempts (12.4% of girls and 7.8% of boys).

The responses to statements about the positive relationships with adults at home and in the community are presented in Table 1, stratified by participant sex. The majority of girls and boys indicated that the three statements about whether parents or other adults at home were interested in schoolwork, believed in their future success, and knew their whereabouts when they were not at home, were either very much true or pretty much true. Similarly, statements about adults outside the home and about friends their own age were reported as pretty much true or very much true by more than half of the participants. Group activities outside home or school were reported at some level by 55–60% of participants, and hobbies such as music and art were reported most often.

Table 1 also shows the proportion of students reporting a past-year suicide attempt for each level of response to statements about relationships in the home and in the community. Suicide attempt prevalence generally decreased with increasing levels of agreement with each statement, indicating that most variables about relationship quality were ordinally related to suicide attempt rates. The only major exceptions to this pattern were in response to the two questions about participation in activities outside the home and school environments such as clubs, sports, and hobbies. For boys, suicide attempts in the past year varied with different levels of engagement in clubs and other group activities, but not in an obvious pattern. Among girls, there appeared to be no relationship between individual hobbies and the prevalence of past-year suicide attempts.

The effect size and statistical significance of each hypothesized predictor variable are shown in Table 2. The measures of positive relationships with adults at home and in the community, and with same-age friends, were significantly and negatively associated with suicide attempts for Hispanic teens of both sexes. The strongest effect size was associated with parents or other adults at home believing in students’ success. For both sexes, each additional level of agreement with the statement was associated with approximately a 50% reduction in the probability of past-year suicide attempts. The effect size of Hispanic teens, who had a parent knowing where they were when they were not at home, was also strong and associated with a decrease in the probability of suicide attempts by nearly 50% with each additional level of agreement with the statement. Positive relationships with community adults had effect sizes that were only slightly lower, indicating a 35–44% reduction in suicide attempt probability. Having caring or helpful friends was also significant, with past-year suicide probability decreasing by approximately 24–34% for each increasing level of endorsement of the two statements about same-age friends.

The involvement in group activities outside of home and school was related to past-year suicide attempt probability for Hispanic girls but not boys. The participation in music, art, and hobbies, was significantly related to past-year suicide attempts for Hispanic boys only. Controlling for age, grades, and parents’ education notably changed the results of only one analysis. For girls, the significant relationship between participation in organized group activities outside the home and school was slightly lower in effect after controlling for confounders (OR = 0.88; 95% confidence interval (CI) 0.79, 0.98; *p*-value 0.018).

Table 3 presents the results of multivariable modeling, including the full model and the final model after reverse stepwise regression. Hispanic boys and girls did not differ with respect to the types of relationships that remained associated with a lower probability of a suicide attempt. Having a parent or other adult at home believing in their success had the greatest effect size, with the probability of suicide attempt reduced by more than 30% for each increased level of agreement with the statement when other variables were controlled. Additionally, related to parents and the home environment, having a parent or guardian know where their teen children were when they were not at home was strongly protective with respect to suicide attempt probability. Importantly, having an adult outside the home or school who tells Hispanic teens when they do a good job remained significant in the model. School grades remained a control variable for Hispanic teens of both sexes and age was a control variable for adolescent girls. The relationships with friends did not remain significantly associated with the probability of past-year suicide attempts when other variables were controlled for Hispanic boys or girls. The F-values for all models, the full and the final models, were significant at *p* < 0.0001.

## 4. Discussion

This study highlights the importance of positive relationships with adults in the home and the community, and the impact they can have on preventing suicide attempts in Hispanic teenagers. Contrary to our hypothesis, the impact of these relationships did not vary by sex. The participation in extracurricular group activities were associated with decreased suicide attempts only for females and did not remain significant when other factors were controlled, suggesting that the benefits from these activities may have been relationships formed with adults rather than the activities themselves. Conversely, hobbies were significantly associated with a reduced probability of suicide attempts only for males. Friendships with peers were significant in bivariate analyses, but not in multivariable modeling. Relationships with adults outside of the home remained significantly protective against suicide attempts in the multivariable model, suggesting that positive relationships with community adults were important to adolescent boys and girls independent of their relationships with their parents.

While the survey results were drawn from adolescents in New Mexico, the results may have had implications for Hispanic adolescents nationally.

### 4.1. Impact of Parents

Our finding that parental support is associated with a lower probability of suicide attempts is consistent with previous research suggesting that conflict with parents is a risk factor for suicide attempts, and that an increased mother–daughter mutuality in Latinas is associated with a decreased risk. As high levels of child–parent conflict are associated with treatment-resistant depression [32], improving the relationships between parents and teens may be especially important in teenagers with a history of previous suicide attempt, suicidal ideation or ongoing depression.

For adolescents of all races and ethnicities who previously attempted suicide, several family-based interventions are proven to be successful in improving the probability of receiving follow-up treatment and preventing a reattempted suicide. Family Intervention for Suicide Prevention is a brief, family-based cognitive behavioral therapy (CBT) session performed in the emergency department (ED) for adolescents presenting to the ED after suicide attempt. It teaches family-based skills for coping with a suicide attempt and safety issues related to preventing a reattempted suicide. The Family Intervention for Suicide Prevention is shown to increase the likelihood of outpatient psychiatric follow up, and thus of ongoing post-attempt psychiatric treatment for teenagers [33]. Another family-focused, ED-based intervention for suicidal adolescents is shown in a randomized controlled trial associated with reduced hospitalization rates, and higher family empowerment and client satisfaction, compared to the usual treatment [34].

Multiple studies found that Safe Alternatives for Teens and Youth, a family cognitive behavioral and dialectical behavioral therapy program, focused on healthy family communication and “extending social ecological systems,” to be more effective than the usual treatment for preventing repeated suicide attempts [35,36]. Parents-CARE is a home-based program for families with adolescents at risk of a suicide attempt that teaches families how to evaluate for and intervene in risky behavior, as well as how to reduce family conflict. While suicide-specific outcomes were not evaluated, parents and youth assigned to the program had high participation rates and rated the intervention as helpful, with parents noting they were still using the skills gained after the program completion [37].

Programs which addressed the specific risk factors for Hispanic adolescents, such as the acculturation gap, and drew on cultural strengths within their family may be of great benefit. *Familias Unidas* is a New York City-based prevention program for substance abuse and risky sexual behavior aimed at Hispanic adolescents. Participants in this program had an improved parent–adolescent communication, and, while there was no specific program content aimed at suicide prevention, adolescents with previously low levels of parent–adolescent communication were found to have a reduced suicide risk after participation [38].

Family-based programs aimed both at suicide prevention and its associated risk factors were also shown to be beneficial and could be broadened to include more teenagers at risk, particularly those without a past history of suicide attempts. Home-based programs may be of particular benefit as they enable program providers to interact with all family members and evaluate family interactions in their natural environment.

### 4.2. Impact of Community

Having a supportive adult in the community was protective against suicide for both Hispanic male and female adolescents, even when controlling for the impact of positive relationships with parents. This is a message that should be spread to communities and community leaders, as well as to parents.

While limited studies exist on the role that community support can play in adolescent mental health, some inroads are made in this field. The Community Partners in Care project focused on adults, but demonstrated that an outreach aimed at addressing factors associated with poverty, such as insecure housing, through community-based settings, such as community centers, barber shops and parks had the ability to improve mental health within the community [39].

For Hispanics, raising community awareness about depression in culturally relevant ways may be a promising community-focused approach. Latino community leaders cite promoting social and cultural connections between Latino adolescents and their communities as important for mental health and suicide prevention [40]. They also pointed to the need for sustainable long-term interventions in mental health care. Hispanic adolescents may be less likely to receive psychiatric care in a traditional setting than their non-Hispanic white peers [41,42], so placing programs targeted at suicide prevention into the community may encourage more people to receive care.

A qualitative study conducted at a community mental health center interviewed suicidal Hispanic adolescents, their parents, and their clinicians [43]. The purpose of the study was to generate an intensive suicide-specific outpatient program for Hispanic adolescents in a community setting. The results of the interviews highlighted the value of a centralized location for bringing people together but also the many barriers faced by families regarding communication, transportation, time, and resources. Most salient were the vastly different perspectives that parents and adolescents had on the severity of suicidal ideation. The program under development seems promising because it was adapted specifically to families with suicidal Hispanic adolescents.

### 4.3. Integration of Community and Family Resources

Given that both community adults and family adults appear to play a protective role, addressing mental health and suicide prevention within a hybrid family–community setting is likely to be most successful.

One example of a promising multisystemic therapy was initially developed for juvenile offenders. This program combined protective factors within an adolescent’s family, school environment and surrounding community. It appeared to be more effective than psychiatric hospitalization in reducing the rates of repeated suicide attempts for adolescents [44]. The study group was predominately African American, but the results could have implications for adolescents of all ethnicities.

Developing family wellness centers in the community and involving successful community adults as “family mentors” to serve as community role models may be additional ways to mobilize community resources and cultural strengths for suicide prevention. Two such family wellness centers with family mentors were completed and showed promise as a model for community engagement in mental health [45].

Life is Precious is a community-based program for Latinas in New York City that addresses Hispanic-specific risk factors such as familism and acculturation. Participants are assessed for suicidal risk factors and family communication and are provided with academic support, wellness education and help with forming positive family relationships. Participation in this program was associated with a decreased suicidal ideation, depression and anger. Given the success of this program, it could serve as an inspiration for other programs that could provide similar opportunities for Hispanic youths [46].

In New Mexico, there is a program that combines features of individual and family counseling, natural helper training, postvention at schools, and community education [47]. The Sky Center of New Mexico Suicide Intervention Project was established in 1994 and currently provides a variety of services remotely (due to COVID-19) and in-person, in English and Spanish. Its counseling and many of its services are free. Several of its programs, such as the program designed to help young Hispanics in crisis avoid rehospitalization, and would be well-suited for a qualitative or quantitative evaluation.

### 4.4. Schools as Place for Intervention, Connection with Family and Community

As positive relationships with adults in a school setting are also associated with a reduced probability of suicide attempt [27], family–community interventions that utilize schools as the intervention point or an additional setting to provide community-based resources may be beneficial. While parent–teacher suicide “gate keeping” programs helped school adults and parents better recognize suicide risks, they were not shown to consistently change behavior [48]. School–parent-based programs were shown to impact risky behaviors associated with suicide, such as substance abuse, as well as decrease conflict with parents [49].

Project Wings Home Visits was a promising home-based mental health program for adolescents and their families that mobilized resources from the public school system, the local university, and a community healthcare system. Community healthcare workers provided home visits for families. Using these community health workers to link school system resources, such as school nurses and mental health providers, with the community-based healthcare system was identified as a priority for participating families [50].

More research is necessary to evaluate the success of these initial community–home hybrid programs, and to address how best to assess the complex impact that family, community, and school environments have on Hispanic adolescent suicide and the ways that adults in these communities might best be mobilized as resources. The programs which integrate family into suicide prevention, with an emphasis on culturally sensitive content, and ensuring that Hispanic adolescents have access to psychiatric resources at school and in the community, should be priorities.

### 4.5. Limitations

Cross-sectional data are, by their nature, limited. The relationships described are measured at a single point in time; therefore, causal inferences are not possible. Therefore, while it is tempting to believe that having parents who are attentive is the reason children are less likely to attempt suicide, it is possible that children who attempt suicide cause their parents to become less attentive. There is no way to know this without longitudinal data.

The self-reported data used in our study came from a New Mexico sample of a national survey of high school students that was administered on a single day. The study sample was limited to student volunteers present in participating schools, who indicated they were Hispanic, and who answered questions regarding past-year suicide attempts and sex. Sex was determined by a question that asked if students were male or female, with no transgender or gender non-conforming options offered.

The survey results may not be generalizable to Hispanic adolescents outside of New Mexico, students who do not conform to traditional gender labels, students who do not regularly attend school, or students who are schooled outside of institutions who participated in the survey, including those who are homeschooled. The survey also excludes Hispanic adolescents lost to suicide, a group to which the protective associations with home and community adults may not apply. The participating Hispanic students come from a wide variety of backgrounds, some domestically born and some immigrants, and important differences in protective factors may be missed when considering all Hispanics as one group.

The survey does not specify which types of different adults in the community or at home students may have relationships with; therefore, it is not possible to draw any further conclusions about how these relationships may be helpful. The associations between suicide and relationships with adults shown to be protective against suicide may be confounded by other, non-measured variables. Furthermore, some of the variables are correlated, so the final models are an approximation. Some of the variables lose their significance due to collinearity and not because of import. Running the models slightly differently could result in a different complement of caring adult behavior that is associated with a reduced probability of suicide attempts.

## 5. Conclusions

Hispanic teens’ suicide attempt risk is reduced by the support of caring adults by as much as 50%. Adult investment in adolescents can be demonstrated in myriad ways: by showing interest in where they are, whether they are doing a good job, or how their schoolwork is going. The quantitative results of our study indicate that the generalized parental interest in, support of, and monitoring of children is protective. For community adults, caring about teens and supporting them should be considered protective when planning community programs. Many promising community-based programs are described in this paper that are either shown to reduce suicide or seem poised to do so. These programs merit imitation, evaluation, and, if effective, support.

## Figures and Tables

**Table 1:** Weighted prevalence of factors hypothesized to be protective with regard to past-year suicide attempts among Hispanic schoolchildren by sex, 2019 NM YRRS.

Statement/Response	Prevalence of Response		Prevalence of Past-Year Suicide Attempt	
	Girls (*n* = 5307)	Boys (*n* = 4734)	Girls (*n* = 5307)	Boys (*n* = 4734)
In my home, there is a parent or some other adult who is interested in my schoolwork				
Not true at all	8.2%	9.4%	23.3%	18.5%
A little true	17.6%	18.5%	17.8%	10.2%
Pretty much true	25.6%	28.3%	11.1%	7.4%
Very much true	48.6%	43.8%	7.5%	3.9%
	100.0%	100.0%		
In my home, there is a parent or some other adult who believes that I will be a success				
Not true at all	4.2%	5.4%	32.0%	22.0%
A little true	9.8%	9.0%	22.1%	21.6%
Pretty much true	18.4%	19.2%	16.4%	10.5%
Very much true	67.6%	66.4%	7.4%	3.4%
	100.0%	100.0%		
When I am not at home, one of my parents/guardians knows where I am and who I am with				
Not true at all	3.8%	6.6%	27.3%	23.2%
A little true	8.0%	10.3%	26.1%	16.4%
Pretty much true	22.7%	26.9%	15.2%	8.0%
Very much true	65.5%	56.2%	7.7%	3.9%
	100.0%	100.0%		
Outside of my home and school there is an adult who really cares about me				
Not true at all	6.9%	8.1%	21.8%	16.3%
A little true	9.8%	9.0%	20.3%	18.8%
Pretty much true	18.4%	18.3%	16.0%	10.6%
Very much true	65.0%	64.6%	8.0%	4.0%
	100.0%	100.0%		
Outside of my home and school there is an adult who tells me when I do a good job				
Not true at all	10.7%	11.5%	20.2%	13.0%
A little true	16.5%	15.4%	19.5%	12.9%
Pretty much true	24.7%	24.0%	10.5%	7.4%
Very much true	48.2%	49.1%	7.6%	3.9%
	100.0%	100.0%		
I have a friend my own age who really cares about me				
Not true at all	5.0%	7.5%	23.6%	18.4%
A little true	9.3%	10.8%	16.6%	11.3%
Pretty much true	17.5%	23.2%	13.0%	6.9%
Very much true	68.3%	58.5%	9.7%	5.7%
	100.0%	100.0%		
I have a friend my own age who helps me when I am having a hard time				
Not true at all	7.2%	10.6%	19.5%	14.4%
A little true	10.6%	14.3%	16.1%	8.9%
Pretty much true	18.5%	23.6%	12.6%	8.2%
Very much true	63.7%	51.5%	9.7%	5.5%
	100.0%	100.0%		
Outside of my home and school, I am part of clubs, sports teams, church/temple or other group activity				
Not true at all	43.3%	39.9%	14.4%	7.4%
A little true	11.6%	13.8%	10.6%	9.4%
Pretty much true	10.3%	11.3%	12.3%	12.4%
Very much true	34.9%	35.0%	8.4%	5.6%
	100.0%	100.0%		
Outside of my home and school, I am involved in music, art, literature, sports or a hobby				
Not true at all	28.2%	24.8%	12.3%	8.6%
A little true	15.4%	13.0%	13.0%	10.0%
Pretty much true	14.0%	14.9%	12.1%	9.2%
Very much true	42.5%	47.2%	10.6%	6.0%
	100.0%	100.0%		

**Table 2:** Probability ratio associated with factors hypothesized to be protective against past-year suicide attempts among Hispanic schoolchildren, by sex, 2019 NM YRRS.

	Girls		Boys	
Resiliency Factor	OR (95% CI)	*p*-value	OR (95% CI)	*p*-Value
Parent/adult at home believes I will be a success	0.54 (0.49, 0.60)	<0.001	0.46 (0.41, 0.52)	<0.001
Parent/guardian knows where I am when not home	0.55 (0.50, 0.61)	<0.001	0.50 (0.43, 0.58)	<0.001
Outside adult who cares about me	0.64 (0.58, 0.71)	<0.001	0.56 (0.50, 0.63)	<0.001
Outside adult who tells me when I do a good job	0.65 (0.60, 0.71)	<0.001	0.56 (0.50, 0.64)	<0.001
Friend my own age who cares about me	0.72 (0.64, 0.79)	<0.001	0.66 (0.56, 0.76)	<0.001
Friend my own age who helps me when I’m having a hard time	0.76 (0.68, 0.84)	<0.001	0.72 (0.63, 0.83)	<0.001
Organized group activities outside home/school	0.83 (0.76, 0.90)	<0.001	0.93 (0.84, 1.04)	0.222
Hobbies (music, art, etc.) outside home/school	0.94 (0.87, 1.02)	0.154	0.87 (0.78, 0.97)	0.013

OR = probability ratio; CI = confidence interval.

**Table 3:** Multivariable models (full model and final model) showing factors associated with lower probability of past-year suicide attempts among Hispanic schoolchildren, by sex, 2019 NM YRRS.

Full Model	F(12, 583) = 13.85		F(12, 562) = 13.63	
	**Girls**		**Boys**	
**Resiliency Factor**	**OR (95% CI)**	***p*-Value**	**OR (95% CI)**	***p*-Value**
Parent/adult at home interested in my schoolwork	0.87 (0.74, 1.0)	0.12	0.88 (0.69, 1.1)	0.26
Parent/adult at home believes I will be a success	0.71 (0.58, 0.87)	0.00	0.62 (0.48, 0.80)	<0.001
Parent/guardian knows where I am when not home	0.74 (0.64, 0.86)	<0.001	0.69 (0.55, 0.88)	0.002
Outside adult who cares about me	0.85 (0.71, 1.0)	0.081	0.92 (0.70, 1.2)	0.54
Outside adult who tells me when I do a good job	0.94 (0.78, 1.1)	0.53	0.78 (0.59, 1.0)	0.078
Friend my own age who cares about me	1.1 (0.82, 1.4)	0.62	0.99 (0.72, 1.4)	0.96
Friend my own age who helps me when I’m having a hard time	1.0 (0.78, 1.3)	0.94	1.3 (0.96, 1.7)	0.10
Organized group activities outside home/school	0.98 (0.87, 1.1)	0.72	1.1 (0.92, 1.2)	0.41
Age *	0.79 (0.70, 0.90)	<0.001	0.93 (0.80, 1.1)	0.37
School grades *	0.75 (0.65, 0.86)	<0.001	0.80 (0.67, 0.96)	0.013
Mother’s education*	1.1 (0.97, 1.2)	0.13	0.98 (0.84, 1.1)	0.79
Father’s education*	1.0 (0.90, 1.1)	0.86	0.93 (0.79, 1.1)	0.40
**Final model**	**F(5, 616) = 41.67**		**F(4, 611) = 46.30**	
	**Girls**		**Boys**	
**Resiliency Factor**	**OR (95% CI)**	***p*-Value**	**OR (95% CI)**	***p*-Value**
Parent/adult at home interested in my schoolwork	__	__	__	__
Parent/adult at home believes I will be a success	0.69 (0.59, 0.80)	<0.001	0.66 (0.55, 0.79)	<0.001
Parent/guardian knows where I am when not home	0.73 (0.64, 0.82)	<0.001	0.72 (0.59, 0.89)	0.002
Outside adult who tells me when I do a good job	0.86 (0.76, 0.98)	0.024	0.76 (0.62, 0.93)	0.007
Age *	0.81 (0.73, 0.90)	<0.001	__	__
School grades *	0.76 (0.68, 0.84)	<0.001	0.79 (0.68, 0.91)	0.001

OR = probability ratio; CI = confidence interval. * control variable.

## Data Availability

The data collection instrument can be downloaded from this site: https://www.youthrisk.org/, (accessed on 30 September 2021). NM-YRRS data can be requested from the New Mexico Department of Health using the Dataset Request Form at: https://www.youthrisk.org/pdf/datarequests/DatasetRequest_Form.pdf, (accessed on 30 September 2021).
